# Power of the Imagination

**Published:** 1863-11

**Authors:** 


Power of the Imagination.—I take from the *Nord* the subjoined narrative, which shows the influence imagination can exercise on the body. That journal vouches for its authenticity. "A gentleman, of great energy of character, while staying at a hotel at Turcoing, was seized with excruciating toothache, accompanied with swelling of the gums. He was recommended to apply a leech; and the keeper of the hotel undertook the operation, using a piece of paper folded up so as to form a kind of case. At the end of a few minutes, as the sufferer did not feel the leech, an examination took place; but the leech was not in the paper, and it had disappeared. It was immediately concluded, to the great horror of the sufferer, that he had swallowed the mordant worm. No sooner was this dreadful conclusion arrived at, than the affrighted man felt terrible bites in his stomach; and, although he swallowed a glass of oil of sweet almonds, renewed bites showed that the leech was still alive. A Doctor was called in, and an ounce of castor oil was administered immediately, but repeated, agonizing pains proved that the leech was in another part of the stomach. A dreadful fear now took possession of the unfortunate man, who anticipated that his intestines would be soon perforated, and that death was certain. A second Doctor called in, prescribed two more ounces of castor oil, which the patient swallowed most stoically, but the bites and pains were renewed. The hotel keeper happened then to change his coat, and in his sleeve he found the leech, which had made its escape at the end of the paper held to the gum of the sufferer. The unfortunate man was thus assured that his life was no longer in danger, but he was a prey to the most uncomfortable sensations from the three ounces of castor oil which he had swallowed."

